# Regulation of IFN-γ-mediated PD-L1 expression by MYC in colorectal cancer with wild-type KRAS and TP53 and its clinical implications

**DOI:** 10.3389/fphar.2022.1022129

**Published:** 2022-12-13

**Authors:** Libin Guo, Xiaoqiong Tang, Sin Wa Wong, Anyuan Guo, Yao Lin, Hang Fai Kwok

**Affiliations:** ^1^ Cancer Centre, Faculty of Health Sciences, University of Macau, Avenida de Universidade, Taipa, Macau SAR, China; ^2^ MoE Frontiers Science Center for Precision Oncology, University of Macau, Avenida de Universidade, Taipa, Macau SAR, China; ^3^ Key Laboratory of Optoelectronic Science and Technology for Medicine of Ministry of Education, College of Life Sciences, Fujian Normal University, Fuzhou, China; ^4^ Central Laboratory at the Second Affiliated Hospital of Fujian Traditional Chinese Medical University, Innovation and Transformation Center, Fujian University of Traditional Chinese Medicine, Fuzhou, China; ^5^ Center for Artificial Intelligence Biology, Hubei Bioinformatics and Molecular Imaging Key Laboratory, Key Laboratory of Molecular Biophysics of the Ministry of Education, College of Life Science and Technology, Huazhong University of Science and Technology, Wuhan, China

**Keywords:** IFN-γ, KRAS, TP53, PD-L1 expression, MYC, anti-PD-1/PD-L1 cancer therapy

## Abstract

**Introduction:** In the tumor microenvironment, interferon gamma (IFN-γ) secreted by tumor infiltrating lymphocytes can upregulate programmed cell death 1 ligand 1 (PD-L1) expression in many cancers. The present study evaluated the expression of PD-L1 in selected colorectal cancer cell lines with IFN-γ treatment and explored the correlation between programmed cell death 1 ligand 1 expression and KRAS/TP53 mutation status.

**Methods:** The selected colorectal cancer cell lines had known KRAS mutations or TP53 mutations. TCGA data analysis were used to investigate the correlation between overall survival of patient with anti-PD-1/PD-L1 immunotherapy and KRAS/TP53 mutation status. Besides, the correlation between PD-L1 expression and KRAS/TP53 mutation status were also investigated by using TCGA data analysis. *In vitro* experiments were used to explore the mechanism underlying KRAS- and TP53-related PD-L1 expression.

**Results:** Firstly, TCGA data analysis for gene expression and overall survival and an *in vitro* study revealed that the wild-type KRAS/TP53 cell lines exhibited hyperresponsiveness to interferon gamma exposure and correlated with better survival in patients receiving anti-PD-1/PD-L1 treatment. Secondly, experimental data revealed that interferon gamma induced the upregulation of programmed cell death 1 ligand 1 mainly through regulating MYC in wild-type KRAS and TP53 colorectal cancers.

**Discussion:** Our findings revealed that the response to anti-PD-1/PD-L1 cancer immunotherapy frequently happened in wild-type KRAS and TP53 colorectal cancers, which were also found to show higher programmed cell death 1 ligand 1 expression. Our results indicate that the wild-type KRAS/TP53 colorectal cancer cell lines may respond better to interferon gamma treatment, which causes increased programmed cell death 1 ligand 1 expression and may be a mechanism underlying the better responses to anti-PD-1/PD-L1 therapies in wild-type KRAS and wild-type TP53 colorectal cancer. Furthermore, the experimental results suggest that interferon gamma regulated programmed cell death 1 ligand 1 expression through the regulation of MYC, which may further affect the response to PD-1/PD-L1 cancer immunotherapy. These results suggest a novel potential treatment strategy for enhancing the efficacy of PD-1/PD-L1 blockade immunotherapy in most colorectal cancer patients.

## Introduction

Colorectal cancer (CRC) is a major cause of mortality and morbidity worldwide (Sung et al., 2021). Colorectal carcinogenesis is closely associated with a variety of genetic and epigenetic changes including mutations in oncogenes, changes in promoters, allelic losses in specific chromosomal arms, tumor suppressor genes, mismatch repair genes, and microsatellite instability (Jass, 2006). Although the overall mortality caused by CRC has declined, survival remains poor. Chemotherapy has been considered as the first therapeutic strategy for the last 20 years, and survival rates have increased with the benefits of targeted therapy (Xie et al., 2020). Currently, the most promising strategy for CRC treatment is immunotherapy. There is a clinical study evaluated the efficacy of pembrolizumab in different subtypes of mismatch repair-deficient cancers (Clinical trial information: NCT01876511). The results of clinical studies have showed promising effects from immune-checkpoint inhibitors (ICIs) in deficient mismatch repair (d-MMR) or highly microsatellite instable (MSI-H) metastatic CRC. Among 32 CRC patients, the objective response rate (ORR) for 10 patients with d-MMR was 40%, while the ORR of MMR-proficient patients was 0% (Le et al., 2017); the disease control rates (DCRs) for the d-MMR and MMR-proficient patients were 90% and 11%, respectively (Le et al., 2017). Based on these results, the US Food and Drug Administration (FDA) approved pembrolizumab in 2017 for the treatment of patients with advanced CRC with MSI-H or d-MMR. However, only 5%–15% of CRC patients are found to have the MSI-H genotype, while most CRC patients have the microsatellite stability (MSS) genotype (Pawlik et al., 2004). It is crucial to search for effective biomarkers beyond MSI status for predicting the response to immunotherapy in colorectal cancer patients. TP53 mutations have been described in approximately 40%–50% of CRC cases (Olivier et al., 2002), and KRAS mutations have been detected in approximately 44% of metastasis CRC patients, with most mutations being identified in codons 12 and 13 of exon 2 (Wong and Cunningham, 2008).

In this study, we investigated the predictive or prognostic role of KRAS and TP53 in CRC treated with anti-PD-1 or anti-PD-L1 therapy and its correlation with PD-L1 expression in CRC. Our data indicate that the KRAS and TP53 mutation status is closely correlated with the response to PD-1/PD-L1 blockade and PD-L1 expression in CRC patients. These results suggest that KRAS- and TP53-related signaling pathways may affect PD-L1 expression, which further influences PD-1/PD-L1 blockade immunotherapy. Determining the underlying mechanism may provide novel strategies for PD-1/PD-L1 blockade immunotherapy. We further found that MYC could affect IFN-γ-induced PD-L1 expression in wild-type KRAS and wild-type TP53 CRC. In summary, our work provides mechanistic insights into why CRC patients with wild-type KRAS and TP53 CRC respond better to anti-PD-1 treatment and highlights that MYC may be an important drug target for improving the efficacy of immunotherapy.

## Materials and methods

### Cell culture, reagents, and plasmid

The human CRC cell lines HCT116 and DLD-1, which harbor a KRAS mutation at codon 13, and their isogenic derivatives lacking mutated KRAS (i.e., HKE-3 and DKS-8), have been previously described (Shirasawa et al., 1993). The HEK293T cell line was obtained from the American Type Culture Collection (ATCC). HKE-3, DLD-1, and DKS-8 were cultured in DMEM/Ham F12 (DMEM/F12) with 10% FBS and 1% (w/v) penicillin/streptomycin. The HCT116 cells were cultured in RPMI 1640 supplemented with 10% FBS and 1% penicillin/streptomycin. The HEK293T cells were cultured in high-glucose DMEM supplemented with 10% FBS and 1% penicillin/streptomycin.

Nutlin-3 was purchased from Sigma. The transcriptional inhibitor actinomycin D was purchased from Beyotime Biotechnology Co., Ltd. Human recombinant IFN-γ was purchased from PeproTech. All the lentivirus plasmids were purchased from OBiO Technology Corporation (Shanghai, China).

### Western blotting

Cells were collected and washed once with cold phosphate buffer saline (PBS). The total cell protein was extracted using RIPA buffer. The protein was quantified using a Pierce BCA Protein Assay Kit, and sodium dodecyl sulfate polyacrylamide gel electrophoresis (SDS-PAGE) was used for protein separation. The wet transfer method was used for protein transfer from the gel to a nitrocellulose membrane. Primary antibodies were diluted using 5% non-fat dry milk in Tris-buffered saline containing Tween 20 (TBST) and incubated overnight at 4°C. The primary antibodies used in this study were anti-PD-L1, anti-MYC, and anti-GAPDH antibodies. The membranes were then washed with TBST three times and incubated with horseradish peroxidase (HRP)-linked secondary antibodies at room temperature for 1 h. Chemiluminescence was detected using the ChemiDoc™ MP Imaging System. The relative expression was quantified with the ImageJ software using GAPDH as an internal control.

### Quantitative real-time polymerase chain reaction (qRT-PCR)

RNA was extracted with the QIAGEN RNeasy^®^ Mini Kit according to the manufacturer’s protocols. cDNA was synthesized using the High-Capacity cDNA Reverse Transcription Kit from 2 μg of total RNA (Applied Biosystems) and then amplified using SYBR Green PCR Master Mix. PCRs were run in a C100 thermocycler (Bio-Rad) using 1 μl of cDNA product. The PCR amplification was performed as per the following steps: incubation for 30 s at 95°C, followed by 40 cycles at 95°C for 5 s and 60°C for 30 s. All the samples were normalized for GAPDH. The primers for the genes studied in this project are provided in Supplementary Table S1.

### Flow cytometry

Cells were treated with IFN-γ for 48 h. Human CD274 was stained with PE-conjugated anti-human CD274 antibody (BioLegend). For groups with 0 ng/ml IFN-γ, values were expressed as fold change of median fluorescence intensity (MFI) vs. HCT116 cells with 0 ng/ml IFN-γ. For groups with 10 or 30 ng/ml IFN-γ, values were expressed as fold change of median fluorescence intensity (MFI) vs. HCT116 cells with 0 ng/ml IFN-γ. All the flow cytometry data were analyzed using the FlowJo software (Treestar Software).

### The cancer genome Atlas (TCGA) patient survival data

The TCGA data were downloaded from the cBioPortal for Cancer Genomics, and the downloaded data included gene expression data, sample data, mutation data, and patient data. We classified the TCGA patients into four subgroups (i.e., KRASwt/TP53wt, KRASmt/TP53wt, KRASwt/TP53mt, and KRASmt/TP53mt) based on the KRAS or TP53 mutation status. Kaplan–Meier survival analyses were used to analyze the association between KRAS/TP53 mutations and survival.

### Lentivirus transduction and cell transfection

Lentivirus-expressing control short hairpin RNAs (shRNAs) or MYC-shRNAs were used in this project (Obio). HCT116 cells or HKE-3 cells were seeded in 6-well plates at a number of 4 × 10^5, and then, the cells were transduced with lentivirus after 24 h. The cells were selected using puromycin 48 h after transduction with lentivirus as mentioned above. In some experiments, the HCT116 and HKE-3 cells were transfected with MYC-shRNA or an MYC-overexpressing plasmid using Lipofectamine 3000 (Thermo Fisher) as per the manufacturer’s protocol and then treated with IFN-γ after 24 h. The cells were harvested for analysis 48 h after IFN-γ treatment.

### RNA sequencing and transcriptome data analysis

Total RNA was isolated using TRIzol reagent (Invitrogen). The RNA integrity was assessed using the RNA Nano 6000 Assay Kit of the Agilent Bioanalyzer 2100 system (Agilent Technologies). Illumina sequencing was carried out at Novogene Bioinformatics Technology Co., Ltd., following the manufacturer’s protocol.

Clean data (clean reads) were obtained, and all downstream analyses were based on high-quality clean data.

The STAR software was used to map clean reads to the human reference genome (GRCh38). The abundance of transcripts directly reflects the gene expression level. The gene expression level was determined from the counts obtained in the sequencing.

### Cancer cells and Jurkat T Cell coculture experiments

The IFN-γ-pretreated cancer cells (i.e., HCT116 and HKE-3) were seeded at 2 × 10^5 cells per well in a 24-well plate and cocultured with activated Jurkat T cells (1 × 10^6 per well). After 24 h, the culture supernatant was collected for the determination of the IL-2 value.

### Transcription factor analysis

The hTFtarget database (http://bioinfo.life.hust.edu.cn/hTFtarget) was used to predict transcription factors of CD274. Subsequently, we also used RNA-seq to explore the expression of TFs in colorectal cancer cells. The JASPAR online software was used to predict the binding sites of the transcription factors near the transcription start size (TSS) of the CD274 DNA sequence.

### Protein-protein interaction (PPI) network analysis

The STRING (https://string-db.org) database, which integrates both known and predicted PPIs, can be applied to predict the functional interactions of proteins. To find potential interactions between DEGs, the STRING tool was employed. The species was limited to “*Homo sapiens*” and an interaction score >0.7 was applied to construct the PPI networks.

### Statistical analysis

The expression data were compared between groups using the Wilcoxon-Mann-Whitney test. The survival curves for each subgroup in the data set were analyzed by the Kaplan-Meier method. The relative mRNA expression was depicted and analyzed using Prism 7 (GraphPad) using the *t*-test method. A *p*-value <0.05 was regarded as significant.

## Results

### KRAS and TP53 status was correlated with PD-L1 expression and anti-PD-1/PD-L1 cancer immunotherapy

**FIGURE 1:**
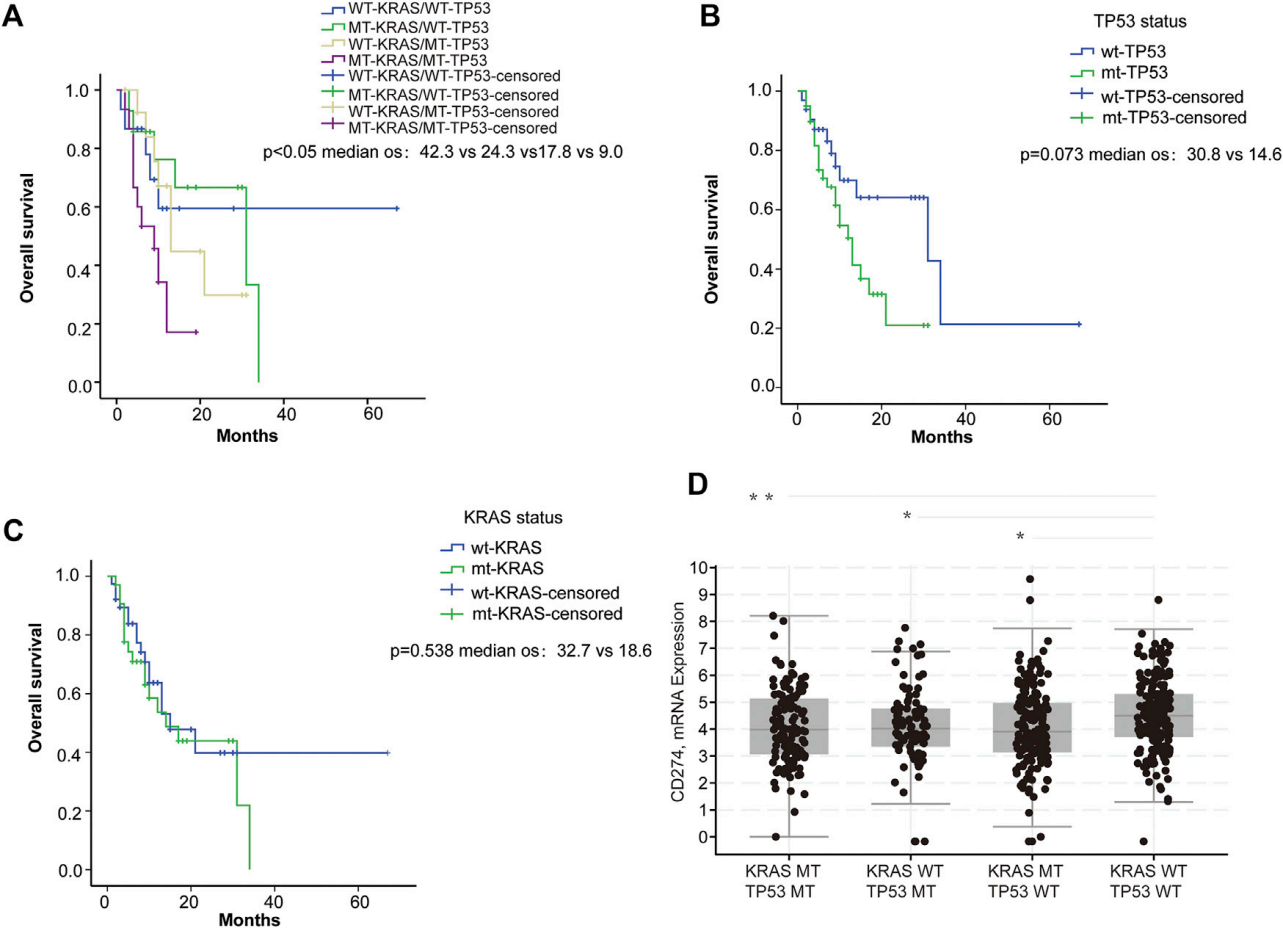
PD-L1 expression was associated with KRAS and TP53 status. **(A–C)** Survival analysis of ICI-treated CRC samples (*n* = 60): **(A)** Survival analysis was used to compare overall survival among the four subgroups di-vided based on KRAS and TP53 mutation status. **(B)** Survival was used to compare overall survival between the wild-type and the mutant TP53 samples; **(C)** Survival analysis was used to compare overall survival between the wild-type and the mutant KRAS samples. **(D)** Analysis of the TCGA colorectal samples (*n* = 594) comparing the differences in CD274 (PD-L1) mRNA expression. *p < 0.05; **p < 0.01.

To explore the potential effect of KRAS and TP53 on anti-PD-1/PD-L1 therapy, we obtained mutation data and patient survival data from the TCGA database. Firstly, we analyzed the correlation between KRAS/TP53 mutations and the survival of patients receiving PD-1/PD-L1 target therapy in TCGA samples from 60 CRC patients. We found that longer survival was shown in patients with wild-type KRAS and wild-type TP53 (Figure 1A), whereas KRAS or TP53 alone did not show significant correlation with patient survival (Figures 1B,C). Especially, patient with wild-type TP53 and wild-type KRAS has longest overall survival time. Next, we further investigated the correlation between KRAS/TP53 mutation and PD-L1 (CD274) expression in TCGA samples from 594 CRC patients. Higher PD-L1 expression was found in patient samples with wild-type KRAS and wild-type TP53 (Figure 1D), compared with expression in patients with mutant KRAS or mutant TP53. Together, results suggested that colorectal cancer patients with wild-type KRAS and wild-type TP53 showed better prognosis and high PD-L1 expression. Publication has also showed that PD-L1 may affect efficacy of anti-PD-1/PD-L1 immunotherapy (Patel and Kurzrock, 2015). Thus, we speculated that the better survival in wild-type KRAS and wild-type TP53 patients was due to the higher PD-L1 expression in those patients.

### IFN-γ-induced PD-L1 expression was correlated with KRAS and TP53 status and inhibited activation of Jurkat T cells

**FIGURE 2:**
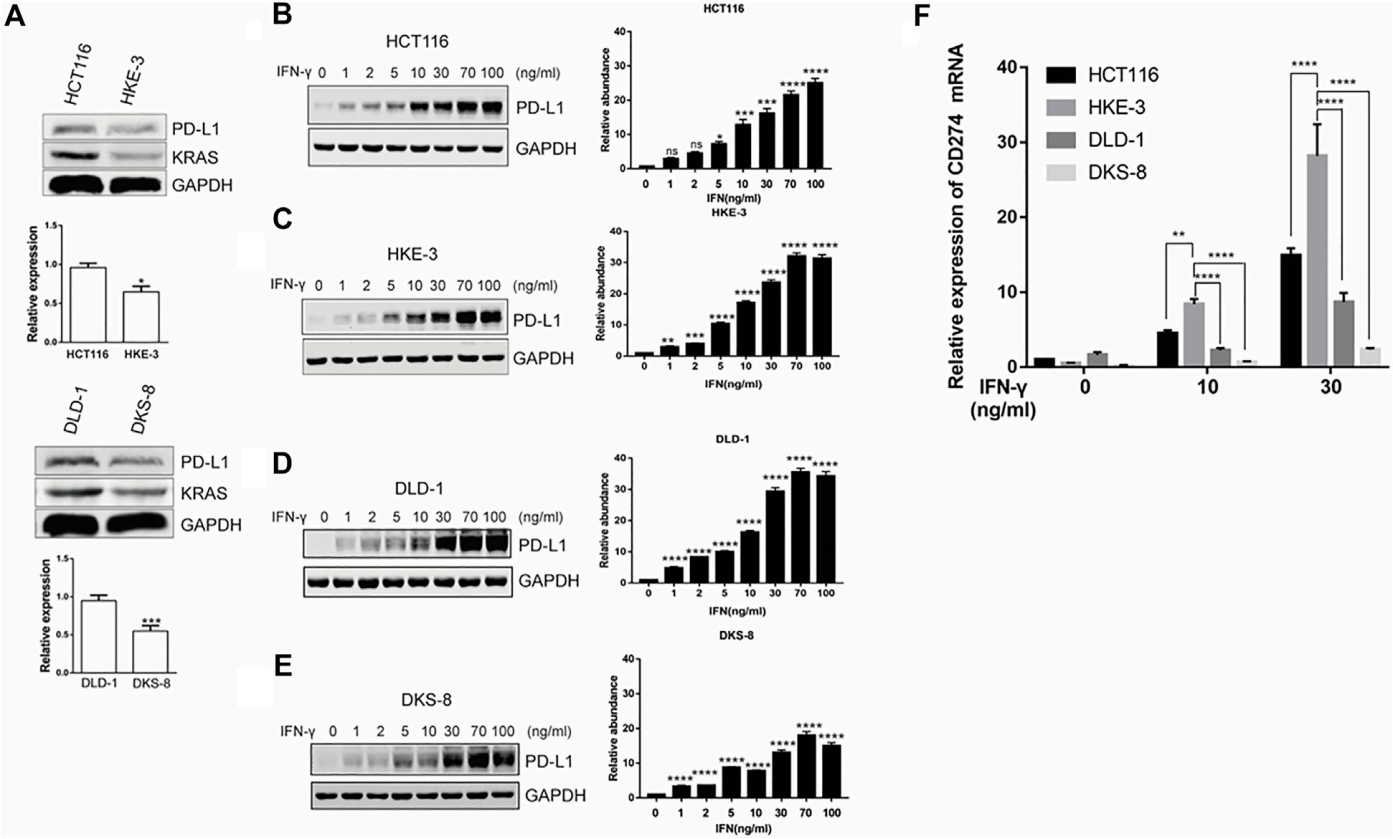
KRAS could affect induced PD-L1 expression in CRC cells (i.e., HCT116 and DLD-1: mutant KRAS cell; HKE-3 and DKS-8: wild-type KRAS cell). **(A)** PD-L1 mRNA and protein in CRC cells were detected using Western blot and RT-PCR analyses. *p < 0.05 vs HCT116; ***p < 0.001 vs. DLD-1 **(B–E)**. IFN-γ induced PD-L1 protein expression in both wild-type KRAS (i.e., HKE-3 and DKS-8) and mutant KRAS cells (i.e., HCT116 and DLD-1) as detected by Western blots. *p < 0.05, **p < 0.01, ***p < 0.001, and ****p < 0.0001 vs. IFN-γ 0 ng/ml **(F)** Higher PD-L1 (CD274) mRNA expression was induced by IFN-γ in wild-type KRAS cells (i.e., HKE-3) among the four CRC cell lines. The four CRC cell lines were treated with different concentrations (i.e., 0, 10, and 30 ng/ml) of human recombinant IFN-γ. After 48 h, cells were collected, and the mRNA expression was determined by RT-PCR. *p < 0.05, **p < 0.01, and ****p < 0.0001 vs. no IFN-γ.

**FIGURE 3:**
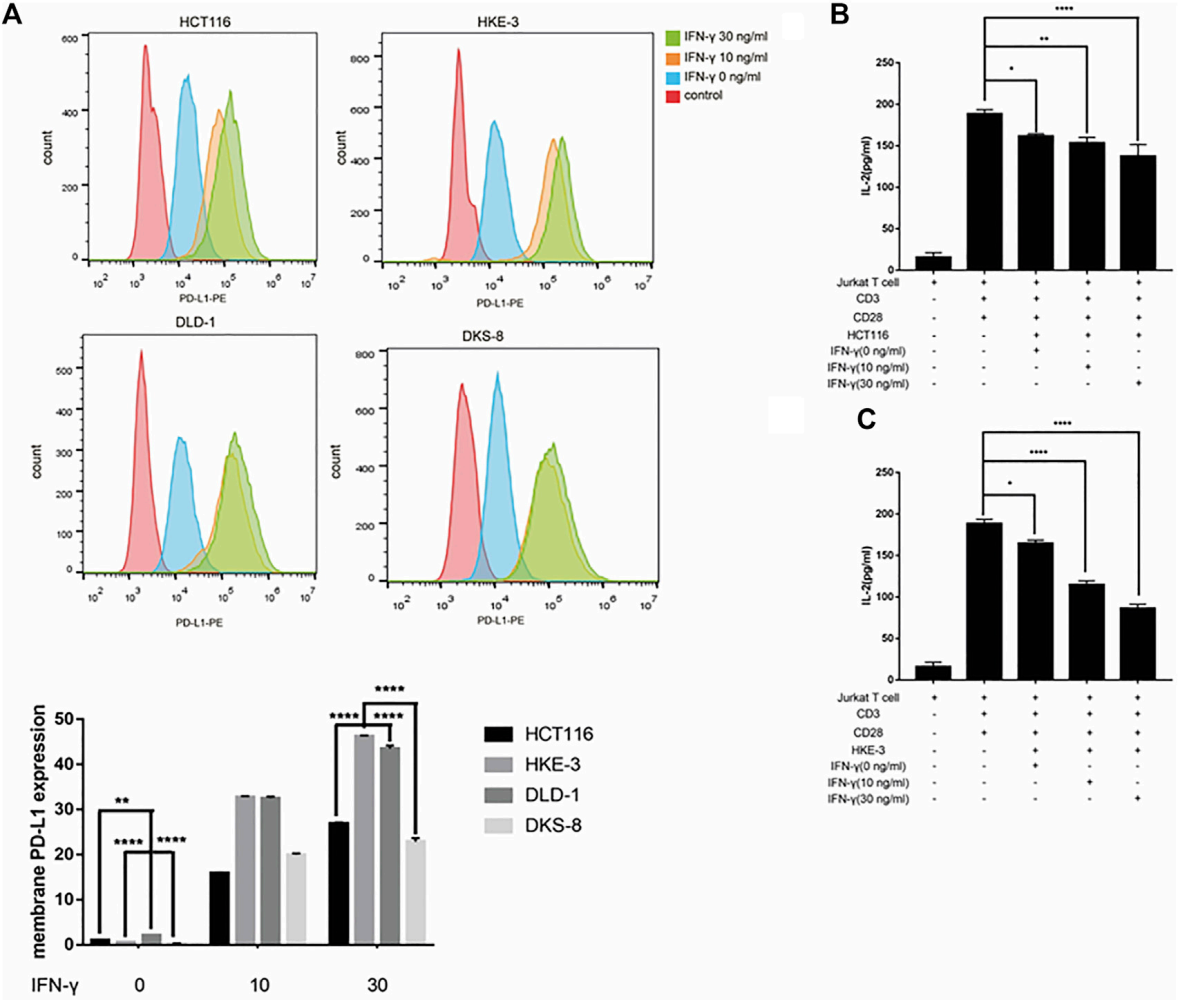
**(A)** Higher PD-L1 expression in membrane was induced by IFN-γ in wild-type KRAS cells (i.e., HKE-3) among the four colorectal cell lines. Surface protein level was detected by flow cytometry. **p < 0.01 and ****p < 0.0001 vs. HCT116 with no IFN-γ. **(B,C)** High PD-L1 induced by IFN-γ in HKE-3 and HCT116 cells inhibited IL-2 production by human Jurkat T cells. *p < 0.05, **p < 0.01, and ****p < 0.0001.

From the database analysis, we found that PD-L1 expression was closely associated with the KRAS and TP53 mutation status in the CRC patient samples. Next, we investigated the effect of the KRAS and TP53 mutation status on PD-L1 expression in CRC cells. In this study, we used two pairs of isogenic CRC cell lines including HCT116, HKE-3, DLD-1, and DKS-8. HCT116 and DLD-1 harbor a KRAS mutation at codon 13, and their isogenic derivatives (i.e., HKE-3 and DKS-8) lack a mutated KRAS. Western blot and RT-PCR analyses showed that the basal PD-L1 expression was higher in the CRC cells with mutant KRAS (i.e., HCT116 and DLD-1) (Figure 2A). When we treated cells with IFN-γ, we first confirmed that IFN-γ could significantly induce PD-L1 expression in different CRC cells (Figures 2B–E). From RT-PCR analysis, we found that higher mRNA levels for PD-L1 were detected in HKE-3 cells compared to HCT116 cells (Figure 2F). Moreover, flow cytometer analysis further showed higher PD-L1 levels in the membranes of HKE-3 cells than those of HCT116 cells (Figure 3A). Most importantly, when coculturing activated Jurkat T cells with CRC cells (i.e., HCT116 and HKE-3), reduced IL-2 secretion was detected when HKE-3 cells were present (Figures 3B,C). These results suggest that HKE-3 cells were more sensitive to IFN-γ and caused higher PD-L1 expression. Furthermore, IFN-γ-induced PD-L1 expression in HKE-3 cells could inhibit the activation of Jurkat T cells. Together, the survival analysis and experimental correlations prompted us to hypothesize that PD-L1 expression induced by IFN-γ is a key mechanism underlying anti-PD-1/PD-L1-mediated immune responses in wild-type KRAS and wild-type TP53 CRC.

### Searching for key regulators participating in the regulation of IFN-γ-induced PD-L1 expression

**FIGURE 4:**
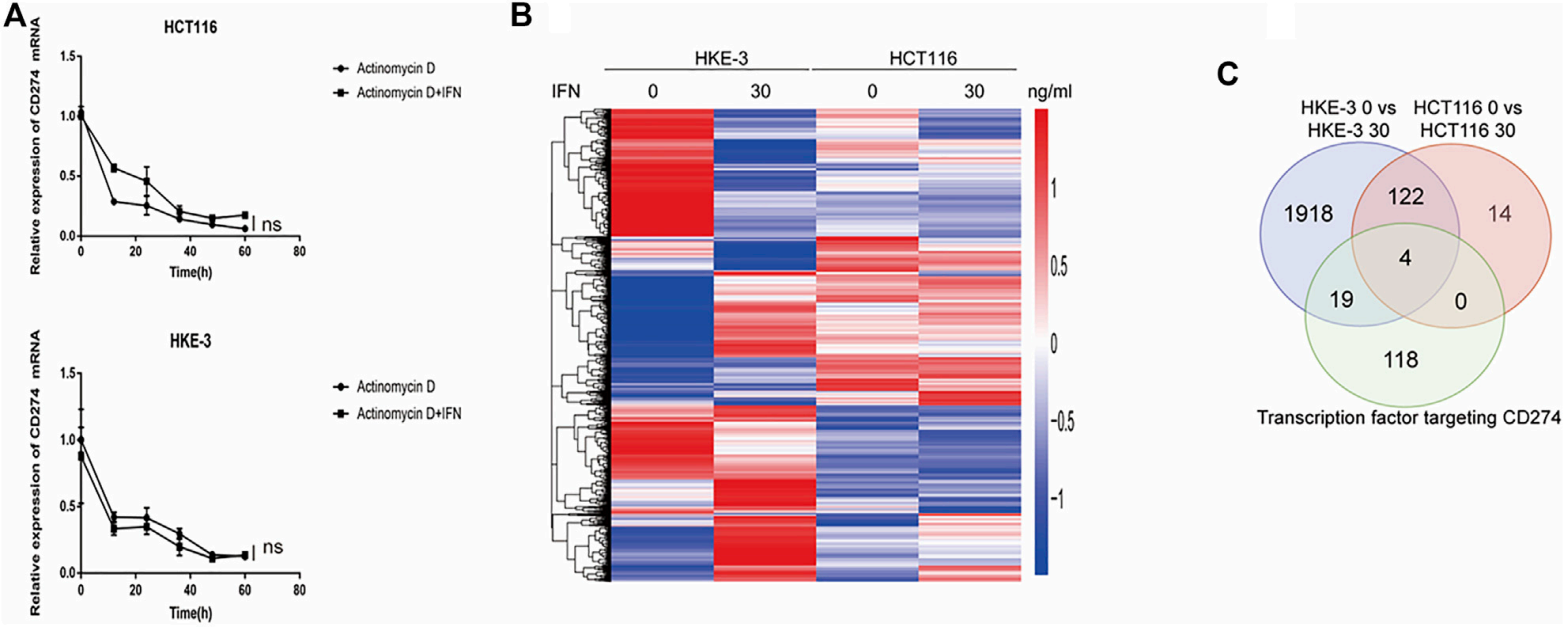
**(A)** Actinomycin D Chase experiments to investigate the effect of IFN-γ on mRNA half-lives in HCT116 and HKE-3 cells. **(B)** Hierarchical clustering of the differentially expressed genes under each experimental condition. Each column represents a sample, and each row represents a gene. The red and blue gradients indicate an increase and a decrease in gene expression abundance, respectively (Liu et al., 2018). **(C)** Venn diagram analysis showing the number of transcription factors targeting the PD-L1 promoter that can be affected by IFN-γ in HCT116 and HKE-3 cells.

**FIGURE 5:**
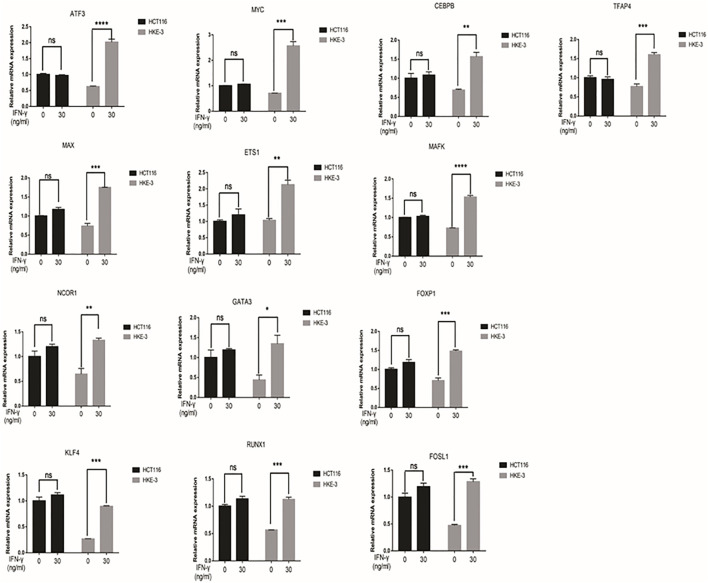
The mRNA expression of selected 13 transcription factors were upregulated by IFN-γ in HKE-3 cells. After being treated with IFN-γ, the mRNA expression of 13 transcription factors in HCT116 or HKE-3 cells was detected by RT-PCR analysis. *p < 0.05, **p < 0.01, ***p < 0.001, and ****p < 0.0001 vs. no IFN-γ.

**FIGURE 6:**
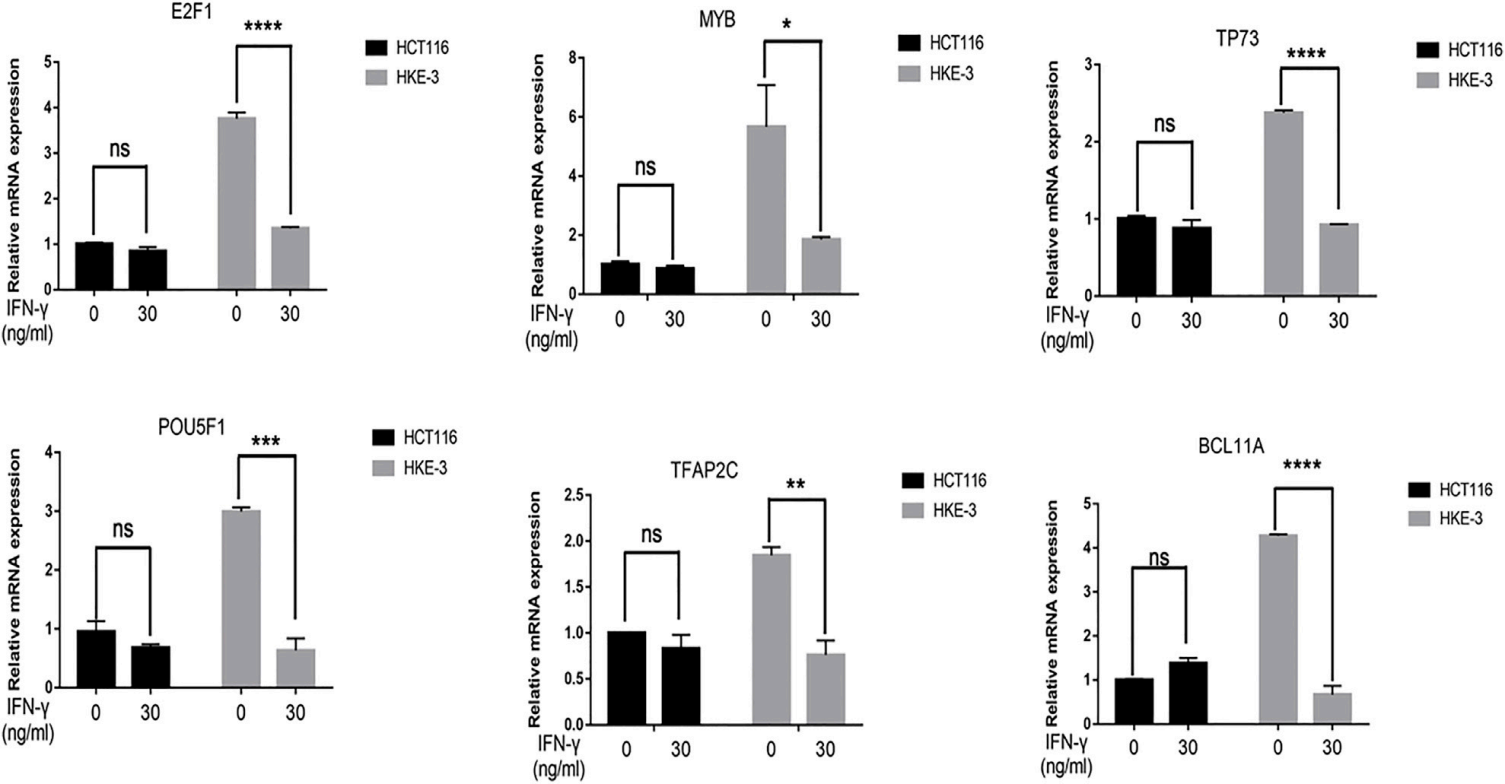
The mRNA expression of selected six transcription factors were downregulated by IFN-γ in HKE-3 cells. After being treated with IFN-γ, the mRNA expression of six transcription factors in HCT116 or HKE-3 cells was detected by RT-PCR analysis. *p < 0.05, **p < 0.01, ***p < 0.001, and ****p < 0.0001 vs. no IFN-γ.

From clinical data and experimental analyses, we found that wild-type KRAS and TP53 CRC cells (e.g., HKE-3 cells) were more sensitive to IFN-γ. This may explain why wild-type KRAS/TP53 patients are more sensitive to anti-PD-1/PD-L1 drugs. IFN-γ can induce PD-L1 by regulating several signaling pathways including the JAK-STAT, PI3K-AKT, and TP53 signaling pathways. To discover the key regulators that are essential for IFN-γ-induced PD-L1 expression in wild-type KRAS and wild-type TP53 cells, we used RNA-seq analysis to determine the genes differentially expressed between HCT116 and HKE-3 cells caused by IFN-γ treatment. Previously, results confirmed that the PD-L1 mRNA expression, total PD-L1 protein level, and membrane PD-L1 protein level were upregulated in CRC cells under IFN-γ treatment. Moreover, the results also show that IFN-γ did not affect the stability of PD-L1’s mRNA (Figure 4A). This means that IFN-γ induced PD-L1 expression through the regulation of mRNA synthesis but not mRNA degradation. Firstly, through transcription factor analysis using hTFtarget database, we have found 141 predicted transcription factors targeting the PD-L1 promoter. Then, through combination of RNA-Seq analysis and use of hTFtarget database, we found 19 transcription factors that were influenced by IFN-γ, and they could bind to the PD-L1 promoter; however, the effect of IFN-γ on the expression of these 19 transcription factors were only found in the HKE-3 cells and not in the HCT116 cells (Figures 4B,C, Figures 5, 6).

### MYC affected IFN-γ-induced PD-L1 expression in wild-type KRAS/TP53 cells

**FIGURE 7:**
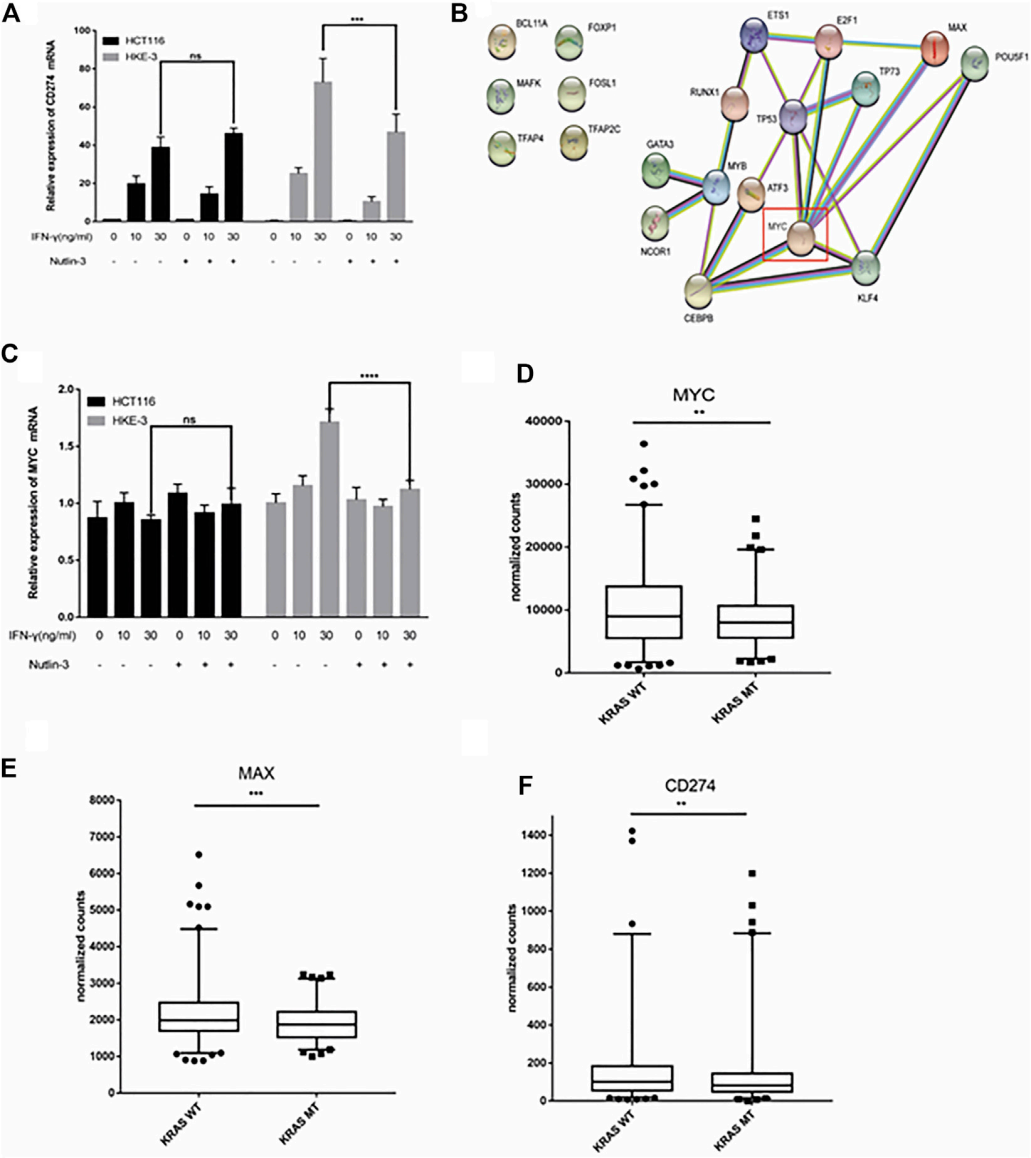
TP53 showed different effects on IFN-γ-induced CD274 expression. **(A)** After treatment with IFN-γ and nutlin-3, cells were collected for detection of CD274 (PD-L1) mRNA expression. **(B)** STRING protein–protein interaction analysis, highlighting the interaction between TP53 and the above selected 19 transcription factors. **(C)** After treatment with IFN-γ and nutlin-3, cells were collected for detection of MYC mRNA expression. **(D–F)** Analysis of CD274, MYC, and MAX gene expression in colon tumor samples with different KRAS mutant statuses using TCGA data. **p < 0.01, ***p < 0.001, and ****p < 0.001.

**FIGURE 8:**
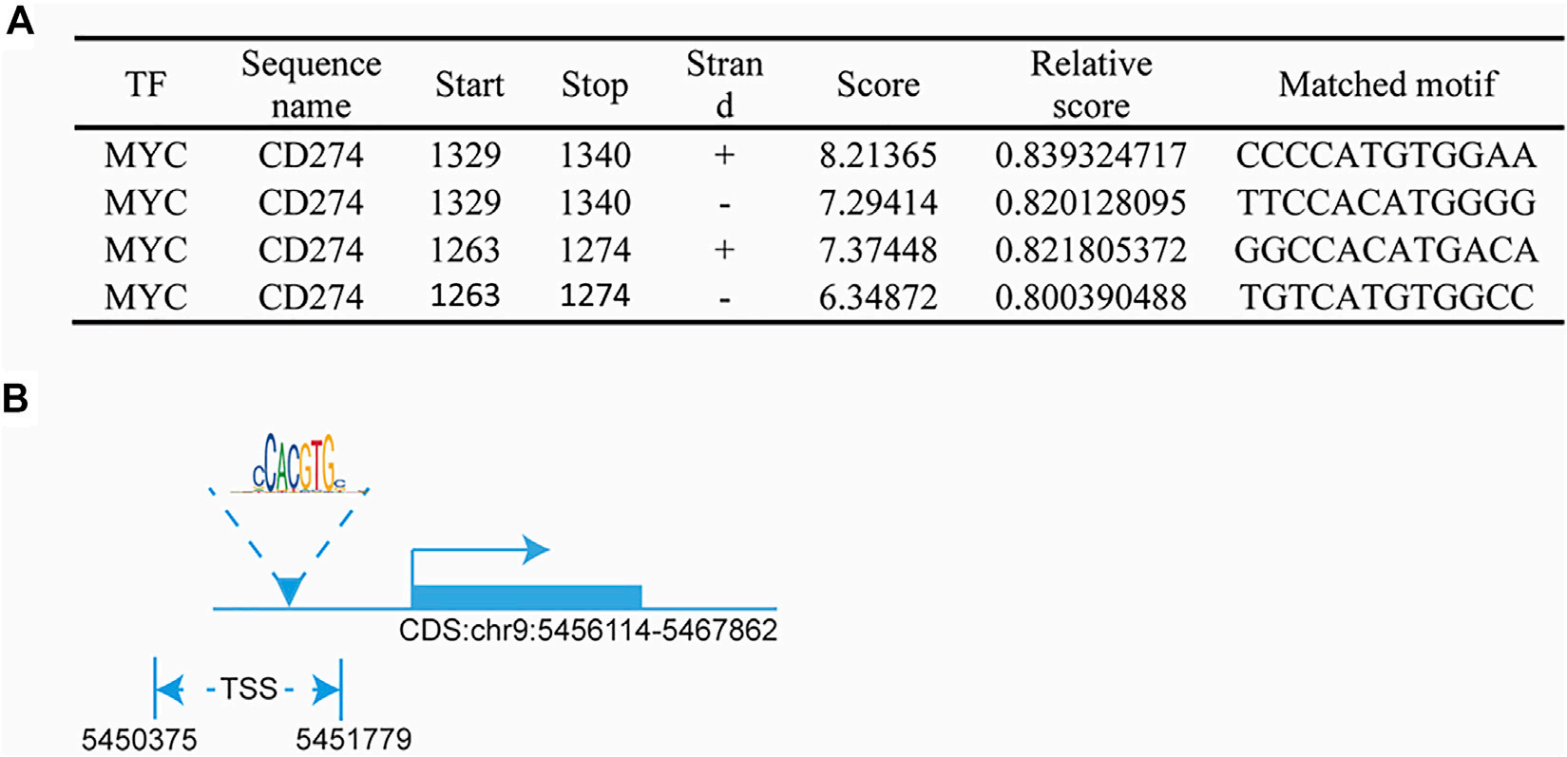
MYC regulated PD-L1 transcription: **(A)** predicted MYC-binding-motif site sequence from the JASPAR 2022 database; **(B)** binding site of MYC around the TSS of the CD274 DNA sequence.

**FIGURE 9:**
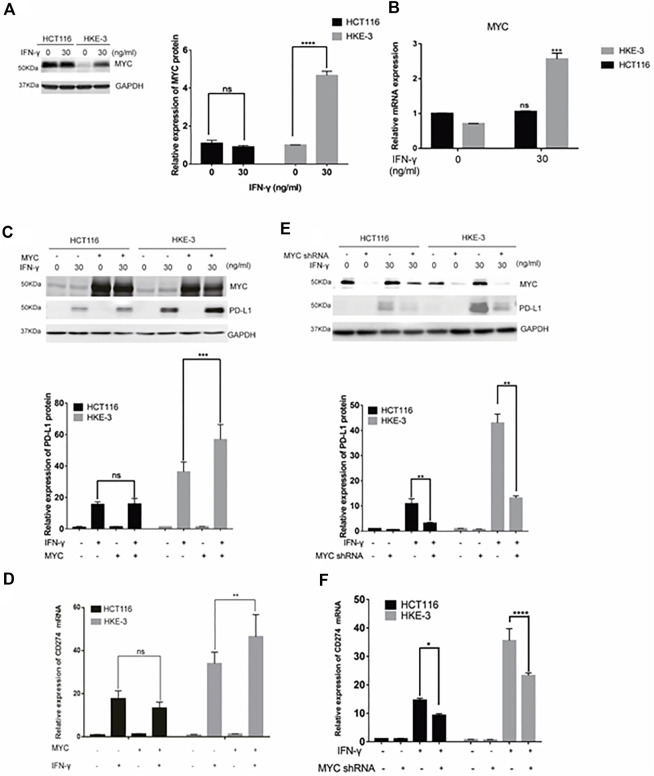
MYC-mediated IFN-γ-induced PD-L1 expression. **(A)** Cells were pretreated with IFN-γ. Then, the cells were collected for Western blot analysis. Treatment with IFN-γ induced MYC expression. **(B)** Cells were pretreated with IFN-γ. Then, the cells were collected for RT-PCR analysis. ***p < 0.001 vs. no IFN-γ. **(C,D)** Cells were transfected with MYC-overexpression plasmid. Then, the cells were treated with IFN-γ. After treatment, cells were collected for Western blot and RT-PCR analyses. MYC overexpression upregulated IFN-γ-induced PD-L1 expression. **(E,F)** Cells were transfected with MYC knockdown plasmid. Then, cells were treated with IFN-γ. After treatment, the cells were collected for Western blot and RT-PCR analyses. Downregulation of MYC decreased IFN-γ-induced PD-L1 expression. *p < 0.05, **p < 0.01, ***p < 0.001, and ****p < 0.0001.

In this study, we found that the different effects of KRAS on IFN-γ-induced PD-L1 expression may be caused by TP53, because HCT116 and HKE-3 cells are isogenic pairs of cells with wild-type TP53, and DLD-1 and DKS-8 cells are isogenic pairs of cells with mutant TP53. Meanwhile, HCT116 and DLD-1 expressed mutant KRAS, and HKE3 and DKS-8 had disrupted mutant KRAS alleles. Then, using nutlin-3 to activate TP53, we found that the activation of the p53 signaling pathway led to the upregulation of induced PD-L1 expression in HCT116 cells (Figure 7A). However, p53 activation caused the downregulation of induced PD-L1 expression in HKE-3 cells (Figure 7A). These results suggest that TP53 shows different effects on induced PD-L1 expression in CRC cells with different KRAS mutation status. In addition, from the protein-protein interaction analysis using the STRING database, we found that MYC is a key regulator among the 19 transcription factors (Figure 7B). The results shown in Figure 7C confirmed that p53 activation caused a decrease in MYC expression in the HKE-3 cells. Moreover, from the gene expression analysis, higher MYC, MAX, and PD-L1 mRNA expression was detected in CRC patients with wild-type KRAS (Figures 7D–F). Potential specific transcription factor binding sites (TFBSs) for MYC were identified around the TSSs of CD274 genes using the JASPAR 2022 database which is shown Figure 8. Then, we further investigated the effect of MYC on IFN-γ-induced PD-L1 expression. Firstly, we found that IFN-γ could regulate MYC expression not only at the mRNA level but also at the protein level (Figures 9A,B). In addition, when we overexpressed the MYC gene in HCT116 and HKE-3 cells, we found that the overexpression of MYC could only upregulate IFN-γ-induced PD-L1 expression in wild-type KRAS cells (i.e., HKE-3) but not in KRAS-MT (i.e., HCT116) cells (Figures 9C,D). When we knocked down the MYC gene in the HCT116 and HKE-3 cells, we found that the downregulation of the MYC gene could also lead to decreased IFN-γ-induced PD-L1 expression in wild-type KRAS cells (i.e., HKE-3) (Figures 9E,F). These results suggest that MYC could affect IFN-γ-induced PD-L1 expression in wild-type KRAS and wild-type TP53 CRC.

## Discussion

In this study, we found that cancer cell lines with the wild-type KRAS and TP53 showed higher IFN-γ-induced PD-L1 expression. Here, wild-type KRAS and wild-type TP53 colorectal cancers responded better to IFN-γ and showed a higher induction of PD-L1 expression. Most importantly, this result was also consistent with the results of clinical analysis—wild-type KRAS and TP53 patients had significantly higher PD-L1 expression levels. In addition, survival analyses based on the TCGA database also showed that patients with wild-type KRAS and TP53 CRC had longer median overall survival when they were treated with anti-PD-1 or anti-PD-L1 antibodies. Recent investigations suggest that IFN-γ could be used to convert cancer cells with a cold tumor environment into those with a hot tumor environment and may be combined with immunotherapy for cancer treatment to improve efficacy (Zhang et al., 2019). This may be due to the increase in PD-L1 expression induced by IFN-γ in the tumor microenvironment. Our study also showed that IFN-γ-treated CRC cells had an inhibitory effect on the activation of Jurkat T cells. These results suggest that IFN-γ could work with anti-PD-1 antibodies in the treatment of wild-type KRAS and TP53 CRC. In our experiment, increased PD-L1 mRNA expression and enhanced protein expression were found in CRC cell lines treated with IFN-γ, which demonstrates that the upregulation of PD-L1 on CRC cell lines may be caused by the activation of the IFN-γ-mediated signaling pathway. Furthermore, our experimental results also suggest that IFN-γ induced PD-L1 expression by regulating mRNA transcription, rather than regulating the translocation of the PD-L1 protein to the cell surface. The results also show that IFN-γ induced the expression of PD-L1 by increasing MYC expression in wild-type KRAS and TP53 CRC cell lines. The role of MYC regarding PD-L1 has been reported in several publications. First, a meta-analysis based on the TCGA database found that PD-L1 expression was closely correlated with MYC, SOX2, and SNAI1 in the endometrial and ovarian cancer data sets (Dong et al., 2018). In addition, MYC was confirmed to function as a transcription factor that could regulate PD-L1 expression by directly binding to the promoters of the PD-L1 gene (Casey, 2016). Moreover, in ESCC (esophageal squamous cell carcinoma) cell lines, ChIP assay results showed that the upregulation of PD-L1 expression was caused by the binding of MYC to the PD-L1 promoter (Liang et al., 2020). Most importantly, it was found that cisplatin induced PD-L1 *via* targeting MYC and could lead to T Cell apoptosis (Sheng et al., 2020). PTEN could also enhance β-catenin/MYC signaling and PD-L1 expression in HBV-expressing hepatoma cells, which leads to decreased PD-1 expression, reduces IL-2 secretion, and induces T Cell apoptosis (Sun et al., 2020). These results suggest that MYC is closely associated with PD-L1 expression and may play a critical role in cancer immunotherapy. Among the 19 transcription factors mentioned above, E2F1 and ATF3 have also shown some correlations with anti-PD-1/PD-L1 immuno-therapy. For example, S1P can upregulate PD-L1 expression through E2F1-mediated transcription and induce T Cell exhaustion (Gupta et al., 2022). In addition, it was found that, among NSCLC patients treated with anti-PD-1 antibodies, lower ATF-3 and PD-L1 expression in tumor tissues from non-responder patients was shown (Liu et al., 2020). In contrast, high PD-L1 expression or high ATF3 expression was correlated with more promising therapy results and improved survival times in NSCLC patients (Liu et al., 2020). This study also showed that ATF3 could directly bind to the TFBS of the CD274 DNA sequence and further regulate PD-L1 expression (Liu et al., 2020). Thus, these two transcription factors may provide some novel strategies for improving the efficacy of anti-PD-1/PD-L1 immunotherapy.

In this project, our results demonstrate that IFN-γ induced PD-L1 expression, which is regulated by MYC in wild-type KRAS and TP53 CRC. Considering the fact that the MYC transcription factor had a positive effect on IFN-γ-induced PD-L1 expression, combination therapies using PD-1/PD-L1 inhibitors and drugs targeting MYC transcription together with IFN-γ could also be potential treatment strategies for wild-type KRAS and TP53 CRC. In summary, our work provides mechanistic insights into why CRC patients with wild-type KRAS and TP53 CRC respond better to anti-PD-1 treatment and highlights that MYC may be an important drug target for improving the efficacy of immunotherapy.

## Data Availability

The original contributions presented in the study are included in the article/Supplementary Material, further inquiries can be directed to the corresponding authors.

## Supplementary material

SUPPLEMENTARY TABLE S1 Primer sequences for real-time PCR.

## References

[B1] Casey S. C. (2016). MYC regulates the antitumor immune response through CD47 and PD-L1 (vol 352, aaf7984, 2016). Science 353 (6296), 229. 10.1126/science.aac9935 PMC4940030 26966191

[B2] Dong P. X. Xiong Y. Yue J. M. Hanley S. J. B. Watari H. (2018). Tumor-intrinsic PD-L1 signaling in cancer initiation, development and treatment: Beyond immune evasion. Front. Oncol. 8, 386. 10.3389/fonc.2018.00386 30283733 PMC6156376

[B3] Gupta P. Kadamberi I. P. Mittal S. Tsaih S. W. George J. Kumar S. (2022). Tumor derived extracellular vesicles drive T cell exhaustion in tumor microenvironment through sphingosine mediated signaling and impacting immunotherapy outcomes in ovarian cancer. Adv. Sci. 9, 2104452. 10.1002/advs.202104452 PMC9108620 35289120

[B4] Jass J. R. (2006). Colorectal cancer: A multipathway disease. Crit. Rev. Oncog. 12 (3-4), 273–287. 10.1615/critrevoncog.v12.i3-4.50 17425506

[B5] Le D. T. Durham J. N. Smith K. N. Wang H. Bartlett B. R. Aulakh L. K. (2017). Mismatch repair deficiency predicts response of solid tumors to PD-1 blockade. Science 357 (6349), 409–413. 10.1126/science.aan6733 28596308 PMC5576142

[B6] Liang M. Q. Yu F. Q. Chen C. (2020). C-Myc regulates PD-L1 expression in esophageal squamous cell carcinoma. Am. J. Transl. Res. 12 (2), 379–388. 32194890 PMC7061834

[B7] Liu H. Kuang X. Zhang Y. Ye Y. Li J. Liang L. (2020). ADORA1 inhibition promotes tumor immune evasion by regulating the ATF3-PD-L1 Axis. Cancer Cell 37 (3), 324–339. 10.1016/j.ccell.2020.02.006 32183950

[B18] Liu Y. Tu Y. Zhang M. Ji G. Wang K. Shan Y. (2018). Identification of molecular pathways and candidate genes associated with cocks’ comb size trait by genome-wide transcriptome analysis. Sci. Rep. 8 (1), 2015. 29386544 10.1038/s41598-018-20373-6 PMC5792444

[B8] Olivier M. Eeles R. Hollstein M. Khan M. A. Harris C. C. Hainaut P. (2002). The IARC TP53 database: New Online mutation analysis and recommendations to users. Hum. Mutat. 19 (6), 607–614. 10.1002/humu.10081 12007217

[B9] Patel S. P. Kurzrock R. (2015). PD-L1 expression as a predictive biomarker in cancer immunotherapy. Mol. Cancer Ther. 14 (4), 847–856. 10.1158/1535-7163.Mct-14-0983 25695955

[B10] Pawlik T. M. Raut C. P. Rodriguez-Bigas M. A. (2004). Colorectal carcinogenesis: MSI-H versus MSI-L. Dis. Markers 20 (4-5), 199–206. 10.1155/2004/368680 15528785 PMC3839332

[B11] Sheng Q. Zhang Y. Wang Z. Ding J. Song Y. Zhao W. (2020). Cisplatin-mediated down-regulation of miR-145 contributes to up-regulation of PD-L1 via the c-Myc transcription factor in cisplatin-resistant ovarian carcinoma cells. Clin. Exp. Immunol. 200 (1), 45–52. 10.1111/cei.13406 31821542 PMC7066384

[B12] Shirasawa S. Furuse M. Yokoyama N. Sasazuki T. (1993). Altered growth of human colon cancer cell-lines disrupted at activated ki-ras. Science 260 (5104), 85–88. 10.1126/science.8465203 8465203

[B13] Sun Y. S. Yu M. X. Qu M. M. Ma Y. H. Zheng D. D. Yue Y. N. (2020). Hepatitis B virus-triggered PTEN/β-catenin/c-Myc signaling enhances PD-L1 expression to promote immune evasion. Am. J. Physiol. Gastrointest. Liver Physiol. 318 (1), G162–G173. 10.1152/ajpgi.00197.2019 31604033

[B14] Sung H. Ferlay J. Siegel R. L. Laversanne M. Soerjomataram I. Jemal A. (2021). Global cancer statistics 2020: GLOBOCAN estimates of incidence and mortality worldwide for 36 cancers in 185 countries. Ca. Cancer J. Clin. 71 (3), 209–249. 10.3322/caac.21660 33538338

[B15] Wong R. Cunningham D. (2008). Using predictive biomarkers to select patients with advanced colorectal cancer for treatment with epidermal growth factor receptor antibodies. J. Clin. Oncol. 26 (35), 5668–5670. 10.1200/Jco.2008.19.5024 19001346

[B16] Xie Y. H. Chen Y. X. Fang J. Y. (2020). Comprehensive review of targeted therapy for colorectal cancer. Signal Transduct. Target. Ther. 5 (1), 22. 10.1038/s41392-020-0116-z 32296018 PMC7082344

[B17] Zhang S. H. Kohli K. Black R. G. Yao L. Spadinger S. M. He Q. C. (2019). Systemic interferon-gamma increases MHC class I expression and T-cell infiltration in cold tumors: Results of a phase 0 clinical trial. Cancer Immunol. Res. 7 (8), 1237–1243. 10.1158/2326-6066.Cir-18-0940 31171504 PMC6677581

