# Supplementary material for: Regulation of IFN-γ-mediated PD-L1 expression by MYC in colorectal cancer with wild-type KRAS and TP53 and its clinical implications
Source: Front Pharmacol. 2022 Dec 13;13:1022129. doi: 10.3389/fphar.2022.1022129 (PMC9792609; doi:10.3389/fphar.2022.1022129)
Supplement: Supplementary file 1 [file Table1.docx]

Table S1. Primer sequences for Real-time PCR

| Gene name |  | Sequence | Product(bp) |
| --- | --- | --- | --- |
| PD-L1 | FW | GCCGACTACAAGCGAATTAC | 234 |
|  | RW | TCTCAGTGTGCTGGTCACAT |  |
| GAPDH | FW | ACATCGCTCAGACACCATG | 143 |
|  | RW | TGTAGTTGAGGTCAATGAAGGG |  |
| MYC | FW | TGAGGAGACACCGCCCAC | 71 |
|  | RW | CAACATCGATTTCTTCCTCATCTTC |  |
| MAX | FW | TGACAAACGGGCTCATCATA | 183 |
|  | RW | TGCTGGTGTGTGGTTTTT |  |
| MAFK | FW | GCACACATGGCAGAGAGAGT | 195 |
|  | RW | GAGTCCTGCTCACCGTCAAA |  |
| KLF4 | FW | TGAACTGACCAGGCACTACC | 110 |
|  | RW | GCCTCTTCATGTGTAAGGCA |  |
| GATA3 | FW | GAACTGTCAGACCACCACAA | 133 |
|  | RW | CTGGATGCCTTCCTTCTTCATA |  |
| TFAP4 | FW | GCAGGCAATCCAGCACAT | 124 |
|  | RW | GGAGGCGGTGTCAGAGGT |  |
| NCOR1 | FW | TCGCTTCCACTGTTTCTGC | 95 |
|  | RW | GGGCTTGACAGCTTCAACTT |  |
| POU5F1 | FW | GTGTTCAGCCAAAAGACCATCT | 156 |
|  | RW | GGCCTGCATGAGGGTTTCT |  |
| MYB | FW | CTACAGCTCAACTCCCTGCC | 284 |
|  | RW | GCATGTGTGGTTCTGTGTTGG |  |
| ATF3 | FW | CCGAGCGGAGCCTGGAG | 271 |
|  | RW | CAGGGGCTACCTCGGCTT |  |
| E2F1 | FW | ACTCCTCGCAGATCGTCATCATCT | 435 |
|  | RW | GGACGTTGGTGATGTCATAGATGCG |  |
| TP73 | FW | AACGCTGCCCCAACCACGAG | 231 |
|  | RW | GCCGGTTCATGCCCCCTACA |  |
| RUNX1 | FW | AGTGGAAGAGGGAAAAGC | 96 |
|  | RW | ATCCACTGTGATTTTGATGG |  |
| TFAP2C | FW | GAAATGAGATGGCAGCTAGG | 267 |
|  | RW | CAGGGTTCATGTAGGATTTG |  |
| ETS1 | FW | TACACAGGCAGTGGACCAATC | 237 |
|  | RW | CCCCGCTGTCTTGTGGATG |  |
| FOXP1 | FW | TCCCGTGTCAGTGGCTATGAT | 226 |
|  | RW | CTCTTTAGGCTGTTTTCCAGCA |  |
| BCL11A | FW | TTCCCTGCGCCATCTTTGTA | 575 |
|  | RW | CCAGTGCAGAAGTTTATCTGCTA |  |
| CEBPB | FW | CAAAACTTTGGCACTGGGGC | 106 |
|  | RW | CATGTGCGGTTGGTTTGGAC |  |
| FOSL1 | FW | CTAAGTGCAGAAACCGAAGAAAG | 110 |
|  | RW | CTTCTGCAGCTCTTCAATCTCTC |  |
